# Gelatin-based anticancer drug delivery nanosystems: A mini review

**DOI:** 10.3389/fbioe.2023.1158749

**Published:** 2023-03-21

**Authors:** Xianchao Jiang, Zhen Du, Xinran Zhang, Fakhar Zaman, Zihao Song, Yuepeng Guan, Tengfei Yu, Yaqin Huang

**Affiliations:** ^1^ Beijing Laboratory of Biomedical Materials, Beijing Key Laboratory of Electrochemical Process and Technology for Materials, Beijing University of Chemical Technology, Beijing, China; ^2^ Beijing Key Laboratory of Clothing Materials R&D and Assessment, Beijing Engineering Research Center of Textile Nano Fiber, Beijing Institute of Fashion Technology, Beijing, China; ^3^ Department of Ultrasound, Beijing Tian Tan Hospital, Capital Medical University, Beijing, China

**Keywords:** gelatin, biomaterials, anticancer, drug delivery, nanosystems

## Abstract

Drug delivery nanosystems (DDnS) is widely developed recently. Gelatin is a high-potential biomaterial originated from natural resources for anticancer DDnS, which can effectively improve the utilization of anticancer drugs and reduce side effects. The hydrophilic, amphoteric behavior and sol-gel transition of gelatin can be used to fulfill various requirements of anticancer DDnS. Additionally, the high number of multifunctional groups on the surface of gelatin provides the possibility of crosslinking and further modifications. In this review, we focus on the properties of gelatin and briefly elaborate the correlation between the properties and anticancer DDnS. Furthermore, we discuss the applications of gelatin-based DDnS in various cancer treatments. Overall, we have summarized the excellent properties of gelatin and correlated with DDnS to provide a manual for the design of gelatin-based materials for DDnS.

## 1 Introduction

Cancer is one of the major causes of global mortality and kills thousands of people each year. The agony and high cost of cancer treatment have wreaked havoc on people’s lives (Siegel et al., 2023). In this case, cancer treatment has become a hot topic of medical research. Anticancer drugs are highly toxic during cancer treatment and have numerous side effects. At the same time, these drugs may not be used effectively. It causes the loss of drugs and may be hazardous to the health of other tissues or organs (Sanchez et al., 2023).

Drug delivery nanosystems (DDnS) can control the drug release rate and targeted area of drug by loading the drug onto a carrier and delivering it into the body (Kong et al., 2023). Superior DDnS are sensitive to different kinds of environmental changes (light, temperature, oxidation, electric, and pH). The carrier allows the drug to remain stable in the body for an extended period and to release continuously for several weeks or months. Drug carriers can also protect human health by reducing the pressure of drugs on the kidneys and reducing the side effects of drugs on the human body through drug controlled release (Jing et al., 2022). With the development of nanotechnology and materials science, several biomaterials have been used to construct DDnS against diseases. For instant, chitosan and its derivatives are often used to treat gastrointestinal diseases, sodium alginate is used to treat inflammatory diseases, and gelatin is used to create targeted and controllable drug-release carriers for cancer and other diseases (Guo et al., 2019; Stagnoli et al., 2023; Yusefi et al., 2023).

Gelatin is a kind of protein molecule hydrolyzed from collagen, which is highly biocompatible and biodegradable under physiological conditions (Coenen et al., 2018). The global gelatin market was valued at USD 5.8 billion in 2021 and is expected to expand at a Compound Annual Growth Rate (CAGR) of 9.5% from 2022 to 2030 (Grand View Research, 2022). It is primarily derived from animals with very low antigenicity, and the metabolites after biodegradation can further contribute to collagen synthesis. Gelatin is a highly responsive material that can respond to several environmental changes which include pH, temperature, and other biological signals (Bhattacharyya et al., 2020; Gheysoori et al., 2023). It has been used as a carrier for drug delivery since 1834, when a patent was granted to Mr. François Achille Barnabe Mothes, a French pharmacy student (Stegemann and Bornem, 1999). Gelatin capsules can control drug dose, effectively improve drug utilization, and enhance the convenience of drug taking and storage capacity (Gullapalli and Mazzitelli, 2017). With the development of nanomaterials, gelatin-based drug delivery carrier materials have developed from macroscopic capsules to DDnS such as gelatin-based nanospheres, hydrogels, nanogels and nanofibers. Gelatin-based drug delivery carrier materials have attracted extensive attention in the field of pharmaceutical medicine (Narayani and Rao, 1995; Fan and Wang, 2016; Liguori et al., 2022; Gheysoori et al., 2023).

**FIGURE 1:**
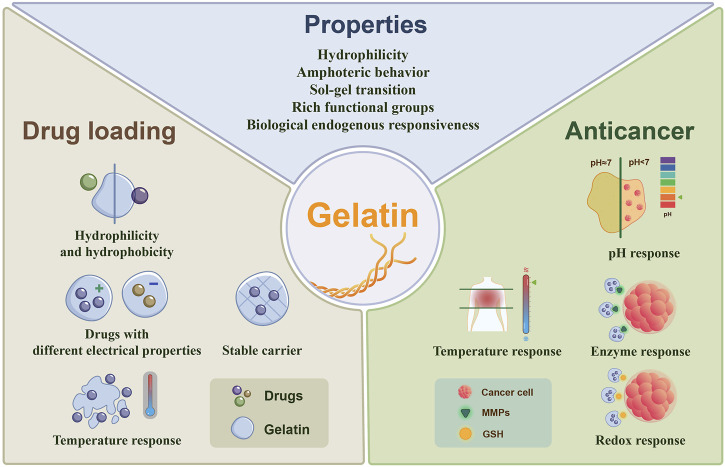
Properties of gelatin used in anticancer DDnS.

Here, we aim to correlate the physicochemical properties of gelatin with anticancer DDnS for different types of cancer treatment (Figure 1). We analyze and summarize the advantages and responsiveness of gelatin as a drug carrier and its specific applications in anticancer drug delivery.

## 2 Properties of gelatin for an anticancer DDnS

### 2.1 Hydrophilic properties

The molecular chain of gelatin has a collagen-like amino acid composition on its surface and contains a high number of hydrophilic groups, which can easily form hydrogen bonds. Thus, gelatin is highly water soluble and can easily bind to drug molecules (Yildirim et al., 2023). Ramanathan et al., used polyhydroxy butyric acid/gelatin nanofibers to release 5-fluorouracil (5-FU) (highly hydrophilic) and methotrexate (MT) (less hydrophilic). They compared the release of two types of drugs having different hydrophilic properties, the highly hydrophilic 5-FU was released first at a higher rate than MT. After 24–96 h, both MT and 5-FU demonstrated a stable release rate that can effectively meet the needs of different types of drugs in the treatment of cancer after surgery (Ramanathan et al., 2022).

By adjusting the hydrophilic properties of gelatin, it can form polymer vesicles or micelles by self-assemble and drug release behavior (Han et al., 2013). Cascone et al. prepared emulsion by dispersing gelatin solution into 25–30 wt% polymethyl methacrylate (PMMA) solution. MT adsorbed on the gelatin surface could be released within 10 h, and the gelatin will swell gradually during the following 30 h, 97% of the loaded MT drug will be released within 10 days (Cascone et al., 2002). Curcio et al. grafted gelatin with polyoxyethylene40 stearate (PEG40ST), which reduced the hydrophilic capacity of gelatin and prepared spherical vesicles systems with narrow size distributions. The content of gelatin controls the release of hydrophilic drug MT from the center of the vesicles. It can effectively reduce the side effects on healthy cells MRC-5 and significantly kill cancer cells H1299 in the lung (Curcio et al., 2015).

Besides, gelatin can be used to modify other anticancer drug delivery carriers to effectively improve the stability *in vivo* by enhancing hydrophilicity. Li et al. developed a gelatin/chitosan/Doxorubicin nanoparticle by using gelatin to crosslink chitosan and loading with Doxorubicin (DOX) (Li et al., 2022). As a nanoshell of the nanoparticles, gelatin improves the loading of DOX effectively and shows good stability *in vivo* compared to chitosan/DOX nanoparticles. It can also enhance the process of drug uptake by cancer cells. Similarly, gelatin can also be used to enhance the stability of graphene materials *in vivo* through hydrophobic interactions on the graphene surface (Hasanin et al., 2022).

### 2.2 Amphoteric behavior/isoelectric point

Gelatin exhibits amphoteric behavior and has different isoelectric points. It has maintained most of the amino acid from collagen during the hydrolysis reaction and contains many functional groups, such as carboxyl amino and hydroxyl groups (Stagnoli et al., 2023). For example, alkaline gelatin (type B) has an isoelectric point at pH = 4.8–5.0 (Narayani and Rao, 1995). In the alkaline environment, Asparagine and Glutamine are converted to Aspartic acid and Glutamic acid. Owing to the presence of amide and carboxyl groups, gelatin is a polyampholyte that exhibits various properties and structures under different pH environments (Bhattacharyya et al., 2020).

Such amphoteric behavior enables gelatin to carry anticancer drugs with different charges. Zou et al. analyzed the loading behavior of DOX, which showed a positive charge and is challenging to load on materials with the same charge (Zou et al., 2013). However, it can be carried on gelatin-based materials as charged amphiphiles. The composites’ hydrophilic and anticancer drug loading capacity is effectively enhanced by using gelatin. Benjamin et al. found a significant decrease in zeta potential after loading DOX to the gelatin-based nanofibers, which confirmed the loading capacity of gelatin for this type of drug (Benjamin et al., 2022).

**FIGURE 2:**
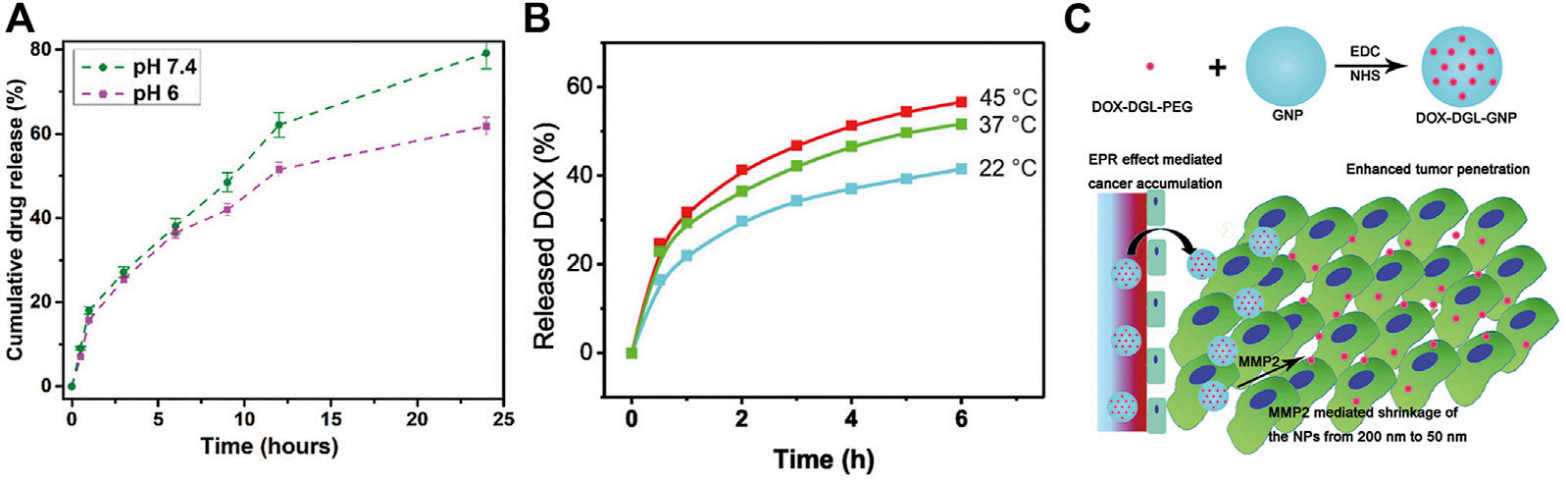
Application of gelatin DDnS in anticancer field. **(A)** The effect of pH on the drug release rate of gelatin DDnS (Bhattacharyya et al., 2022); **(B)** The effect of temperature on the drug release rate of gelatin DDnS (Suarasan et al., 2016); **(C)** Application of MMP-2 responsiveness of gelatin in anticancer DDnS (Hu et al., 2015).

Besides, gelatin is a highly responsive molecule. The morphology of gelatin will transform after perceiving the changes in the environment. When the pH of the environment is far away from the isoelectric point, gelatin converts to linear structure and tends to dissolve in water. Additionally, the gelatin nanocarrier material converts to different charge and creates an electrostatic repulsion with the drug, thus releasing the drug into the cell interior (Ozdabak-Sert et al., 2019). Bhattacharyya et al. prepared a DDnS using gelatin nanoparticles and loaded with 5-FU. This DDnS has a selective and sustained release profile in the pH range 6–7.4, which can counteract some of the drawbacks of 5-FU administration (short duration of action and poor specificity) (Figure 2A) (Bhattacharyya et al., 2022).

### 2.3 Sol-gel transition

Gelatin is a kind of thermo-sensitivity material that can strongly respond to heat, typically as a sol-gel transition (Gheysoori et al., 2023). However, it is necessary to adjust the temperature range of gelatin sol-gel transition to control the anticancer drug release by thermal response (Chen et al., 2011). Slemming-Adamsen et al. prepared nanofibers with gelatin and Poly (N-isopropylacrylamide (PNIPAM), which exhibit thermo-responsive swelling/deswelling function. When loaded with the anticancer drug DOX, the fibers can significantly release DOX according to the temperature increase, effectively reducing the viability of human cervical cancer cells Hela (Slemming-Adamsen et al., 2015). Moreover, temperature is one of the most widely used trigger signals for DDnS in all response types. Various temperature-responsive drug carriers are often used as an adjunct to cancer treatment (Nardecchia et al., 2019). A temperature-responsive DOX gelatin-based nanodrug delivery carrier has been developed (Figure 2B) (Suarasan et al., 2016).

### 2.4 Crosslinking of gelatin

The extreme water solubility may also cause the gelatin microspheres to dissolve before drug delivery. Crosslinking can improve the stability of nanomaterials for carriers and control the release of drugs. It can be classified as physical, enzymatic, and chemical methods. The reaction sites for gelatin-based material DDnS are mostly amino and carboxyl groups. Typically, non-toxic genipin or oxidized polysaccharide-based natural polymers/extracts are selected for crosslinking reactions. In an earlier study, Fan et al. utilized a less toxic glutaraldehyde crosslinked gelatin. The higher glutaraldehyde content reduces the rate of DOX release, but it will not affect the stability of DOX *in vivo* (Fan and Dash, 2001).

Lately, Liguori et al. prepared a highly biocompatible gelatin-hydrogel composite, which was crosslinked by genipin and loading with anticancer drug Chlorotetracyline hydrochloride (CTC) (Liguori et al., 2022). The crosslinking of genipin has little effect on the CTC release behavior of gelatin hydrogels. Meanwhile, the gelatin hydrogel layer slows down the release rate of the Diclofenac drug from the inner nanofibers. The drug release rate of the composite can be controlled by regulating the thickness of the hydrogel to satisfy the requirement of the long-term release of anticancer drugs. Besides, by grafting with methacryloyl groups, gelatin can be turned into photo-crosslinked materials. After UV light irradiation, both photo-chemical and cross-linking reactions occur, which is suitable for hydrogel DDnS preparation for minimally invasive cancer treatment (Zhou et al., 2022).

### 2.5 Other properties

As a collagen hydrolysate, gelatin will also respond to other endogenous biological signals, such as enzymes and redox (Zhou et al., 2020). Matrix Metalloproteinases (MMPs) are overexpressed in tumor cells and can break collagen and other similar amino acid sequences (Jabłońska-Trypuć et al., 2016). The enrichment of MMP-2 in the tumor cell region promotes the drug-release effect of the gelatin-based carrier material in the tumor region (Vaghasiya et al., 2021; Li et al., 2022).

## 3 Application for different kinds of cancer

### 3.1 Breast cancer

Breast cancer is one of the leading causes of death in women worldwide. Uncontrolled proliferation of breast epithelial cells enabling the use of both intrinsic/chemical stimuli (e.g., pH) and extrinsic/physical stimuli (e.g., heat) as stimulus response signals for drug carriers in therapy. Gelatin is an ideal preparation material for drug delivery in the treatment of breast cancer.

The pH-responsive gelatin-coated gold nanoparticle MT DDnS was prepared by Khodashenas et al. The results showed that MT was released from the carrier at two different pH values (5.4 & 7.4) at different rates. The highest drug release rate was observed at pH 5.4, which makes this drug delivery system suitable for use in breast tumor environments. Furthermore, gelatin-based nanodrug-loaded particles also improved the efficacy of MT, indicating a synergistic effect between the drug carrier and the drug (Khodashenas et al., 2021).

Because the focal area of breast cancer is susceptible to external stimulation, cancer hyperthermia becomes a high-efficiency treatment tool. Derakhshankhah et al. developed a pH, temperature dual-responsive drug delivery nanogel for cancer hyperthermia. The hydrogel collapse at 42°C, allowing the drug to be released more efficiently. The charge of the hydrogel is opposite to that of the anticancer drug DOX, thus DOX can be successfully loaded into the hydrogel. The drug release rate of the hydrogel at pH 4.2 (64.7%) is significantly higher than that at pH 7.4 (25.7%) due to the strong hydrogen bonding and ionic interactions between the drug molecules and the polymer network (especially gelatin) at neutral pH, in contrast to the amine-based protonation occurring in gelatin at acidic pH which facilitates the release of the drug (Derakhshankhah et al., 2021).

### 3.2 Lung cancer

Lung cancer is one of the most commonly diagnosed cancers, with approximately two million new cases and 1.76 million deaths each year. The inhalation route of drug delivery is a therapeutic modality unique to lung cancer treatment that enables the delivery of drugs directly to the lungs, which can greatly reduce the side effects and improve the efficacy of the drug. Gelatin drug carriers have been used clinically for aerosol delivery.

Abdelrady et al. prepared gelatin nanoparticles of suitable size for lung cancer cell uptake and higher cancer cytotoxic efficiency by designing and optimizing the preparation parameters of gelatin nanoparticles. MT was chemically coupled to gelatin, and the positive net charge of gelatin at a healthy human environmental pH was more favorable for binding to MT. The nanoparticles exhibit good nebulization properties and lung deposition in the bronchial tract and are well suited to release anticancer drugs when stimulated by the lung cancer microenvironment (Abdelrady et al., 2019).

### 3.3 Colorectal cancer

Fluoropyrimidines are the main chemotherapeutic agents used in the treatment of colorectal cancer, and they exert their antitumor activity by disrupting the synthesis and function of DNA and RNA. These drugs can be administered orally for the treatment of colorectal cancer, which is more convenient, flexible, and acceptable for patients, but may be affected by the gastrointestinal environment (Kwakman and Punt, 2016; Garcia-Alfonso et al., 2021). Therefore, the use of oral drugs for the treatment of colorectal cancer requires many methods to protect the drugs from gastrointestinal pH and enzymatic degradation and then to ensure controlled release in the proximal colon (Vinchhi and Patel, 2021).

Anirudhan et al. prepared a 5-FU pH-responsive hydrogel drug carrier by crosslinking β-cyclodextrin grafted gelatin with oxidized dextran. It was found that β-cyclodextrin grafting increased the drug encapsulation capacity, and pH-sensitive swelling and degradation contributed to drug release into the colon. The drug release depended on the pH of the medium, demonstrating that the prepared hydrogel drug carriers could release the drug selectively in the basal environment of colonic, and rectal mucosa. And the increase in gelatin dosage also increased the drug loading rate of hydrogel drug carriers (Anirudhan and Mohan, 2014).

### 3.4 Skin cancer

Transdermal DDnS is a commonly used strategy for skin cancer treatment. However, due to poor penetration of the drug into the stratum corneum and low efficacy, higher drug concentrations are often required to achieve the therapeutic effect, which causes many adverse effects on the skin and reduces the utilization of the drug (Jain et al., 2020). Skin cancer is a malignant lesion of skin cells and thus accessible to external stimuli such as light and electro current. Combined with responsive drug carriers, this strategy can be used to overcome drug delivery barriers in skin cancer treatment.

Oktay et al. prepared electro-responsive hydrogels by doping poly (3,4-ethylenedioxythiophene)/poly (styrenesulfonate) (PEDOT/PSS) into gelatin methacryloyl. PEDOT/PSS has good electrical conductivity and is uniformly dispersed in the gelatin polymer network, and PSS also provides continuous transport for electron paths. Based on the electrical responsiveness of the prepared conductive gelatin, the hydrogel shows an increased release of the loaded anticancer drug 5-FU when an external electric field is applied, which can be used as a drug carrier for the controlled release of electrical stimulation for the treatment of skin cancer (Oktay and Alemdar, 2019).

### 3.5 Other cancers

In the microenvironment of many different types of cancer, redox disorders and disruption of enzyme levels occur. MMPs are overexpressed in focal areas of many cancers (Deng et al., 2019; Kicman et al., 2022). Glutathione (GSH) is similarly overexpressed in these cancer lesion areas (Nunes and Serpa, 2018; Huang and Zhan, 2022). Several responsive drug carriers have been developed using enzymatic and redox disorders.

Zhou et al. prepared a redox- and MMP-2-sensitive gelatin nanoparticle drug carrier by grafting the anticancer drug paclitaxel (PTX) onto sulfhydryl modified gelatin through disulfide bonds. Overexpressed GSH in cancer can rapidly break the disulfide bond, and MMP-2 overexpressed in cancer can degrade the gelatin carrier, and through these two pathways, PTX can be released precisely and efficiently into the cancer lesion area (Zhou et al., 2020). This type of response is suitable for application to the majority of cancer drug release carriers. Fan et al. designed a spatially controlled multistage nanodrug carrier by encapsulating polyamidoamine (PAMAM) dendrimers in gelatin nanoparticles. The MMP-2 responsive drug release mode brought by gelatin as a drug carrier can effectively prevent the leakage of the anticancer drug MT from the core of the PAMAM to the external non-focal environment (Fan et al., 2017). Similarly, Hu et al. loaded DOX onto Dendritic poly-Lysine (DGL) and conjugated it on the surface of gelatin nanoparticles to construct an MMP-2 sensitive anticancer DDnS (Figure 2C) (Hu et al., 2015).

## 4 Outlook

Gelatin is a kind of drug carrier material with great potential and responsiveness to cancer treatment, which can be used for targeted drug delivery to cancer sites. At the same time, the combination of gelatin drug carriers with hyperthermia can effectively reduce the number and bioactivity of cancer cells and inhibit the proliferation of cancer cells. In this case, responsiveness becomes particularly important, and preparing responsive materials requires the matrix to be inherently capable. Gelatin is a reliable carrier material with outstanding responsiveness. Besides, gelatin has been used in other fields, such as targeted drug delivery capsules dissolved in the stomach or intestine, adhesive for wound repair, and nanogels for drug release in the central nervous system or through the skin (Duconseille et al., 2015; Yuk et al., 2019; Augustine et al., 2021). As a natural biological material, gelatin has a broad application prospect in the medical field. The source of gelatin will significantly affect the properties of gelatin, but not everyone recognizes mammalian gelatin or gelatin products without detailed source information because of socio-cultural and religious issues.

In this mini-review, we highlight the main advantages of gelatin as a carrier for anticancer drugs and preparation methods to develop DDnS using its responsiveness. By exploiting the responsiveness of gelatin, it can provide significant inspiration for cancer treatment and the preparation of new materials.
